# Association of bacterial genotypes and epidemiological features with treatment failure in hemodialysis patients with methicillin-resistant *Staphylococcus aureus* bacteremia

**DOI:** 10.1371/journal.pone.0198486

**Published:** 2018-06-04

**Authors:** Shang-Yi Lin, Hung-Pin Tu, Tun-Chieh Chen, Mei-Chiou Shen, Yi-Wen Chiu, Yen-Hsu Chen, Po-Liang Lu

**Affiliations:** 1 Division of Infectious Diseases, Department of Internal Medicine, Kaohsiung Medical University Hospital, Kaohsiung, Taiwan; 2 School of Medicine, Graduate Institute of Medicine, College of Medicine, Sepsis Research Center, Kaohsiung Medical University, Kaohsiung, Taiwan; 3 Department of Public Health and Environmental Medicine, College of Medicine, Kaohsiung Medical University, Kaohsiung, Taiwan; 4 Kaohsiung Municipal Ta-Tung Hospital, Kaohsiung Medical University Hospital, Kaohsiung Medical University, Kaohsiung, Taiwan; 5 Department of Pharmacy, Kaohsiung Medical University Hospital, Kaohsiung Medical University, Kaohsiung, Taiwan; 6 Department of Internal Medicine, Kaohsiung Medical University Hospital, Kaohsiung, Taiwan; 7 Department of Laboratory Medicine, Kaohsiung Medical University Hospital, Kaohsiung, Taiwan; Chang Gung Memorial Hospital, TAIWAN

## Abstract

**Objectives:**

Methicillin-resistant *Staphylococcus aureus* (MRSA) infections in the hemodialysis (HD) population are epidemiologically classified as healthcare-associated infections. The data about the clinical impact and bacterial characteristics of hospital-onset (HO)- and community-onset (CO)-MRSA in HD patients are scarce. The current study analyzed the difference in the clinical and molecular characteristics of HO-MRSA and CO-MRSA.

**Methods:**

We performed a retrospective review and molecular analysis of clinical isolates from 106 HD patients with MRSA bacteremia from 2009 to 2014. CA genotypes were defined as isolates carrying the SCC*mec* type IV or V, and HA genotypes were defined as isolates harboring SCC*mec* type I, II, or III.

**Results:**

CO-MRSA infections occurred in 76 patients, and 30 patients had HO-MRSA infections. There was no significant difference in the treatment failure rates between patients with CO-MRSA infections and those with HO-MRSA infections. CA genotypes were associated with less treatment failure (odds ratio [OR]: 0.18; 95% confidence interval [95% CI], 0.07–0.49; *p* = 0.001). For isolates with a vancomycin minimum inhibitory concentration (MIC) < 1.5 mg/L, the multivariate analysis revealed that HA genotypes and cuffed tunneled catheter use were associated with treatment failure. For isolates with a vancomycin MIC ≥1.5 mg/L, the only risk factor for treatment failure was a higher Pitt score (OR: 1.76; 95% CI, 1.02–3.05; *p* = 0.043).

**Conclusion:**

CA genotypes, but not the epidemiological classification of CO-MRSA, impacted the clinical outcome of MRSA bacteremia in the HD population.

## Introduction

Although there has been a decreasing trend in the incidence of methicillin-resistant *Staphylococcus aureus* (MRSA) infections in the past decade [1], MRSA remains a major threat to patients all over the world. The hemodialysis (HD) population has a high risk for invasive MRSA infections that is more than 100-fold higher than the incidence rate of the general population (45.2/1000 *vs*. 0.2–0.4/1000) [2]. The incidence of invasive MRSA infections among dialysis patients decreased by 7.3% annually during 2005–2011 in the USA [3]. However, a rate of 19.9% for healthcare-associated invasive MRSA infections was identified among dialysis patients in the USA in 2014 [4]. MRSA infections in HD patients are associated with long hospitalizations, high costs and mortality [5]. Therefore, the impact of MRSA infections in the HD population remains significant.

All MRSA infections in the HD population are classified as healthcare-associated (HA) infection according to epidemiologic classification. HA-MRSA infection can be further classified as healthcare-associated hospital-onset (HO) or healthcare-associated community-onset (CO) infections based on infection development in the hospital or community [4]. According to molecular classification, MRSA strains are classified as HA- or community-associated (CA)-MRSA strains by genotyping. Strains carrying SCC*mec* element type I, II or III are defined as HA genotypes, and those harboring SCC*mec* type IV or V are defined as CA genotypes [6, 7]. The high prevalence and disease burden of CA-MRSA have been increasingly observed in many countries [8 – 10]. Furthermore, CA genotypes are increasingly implicated in nosocomial infections and may eventually displace HA genotypes in hospitals to become the predominant nosocomial MRSA isolates [11]. A population-based study in the USA showed the proportion of CA genotypes increased among chronic dialysis patients [3]. However, very little is known regarding whether these differences are associated with the clinical outcomes of MRSA bacteremia among the HD population [3]. We aimed to investigate the epidemiologic differences, clinical presentations, molecular characteristics and outcomes in HD patients with MRSA bacteremia in this study.

## Materials and methods

### Study design and patient population

This retrospective study was conducted in Kaohsiung Medical University Hospital, a 1600-bed medical center in Taiwan, after approval was received from the institutional review board (IRB No. KMUH-IRB-990139). The medical records of HD patients with MRSA bacteremia from January 2009 to October 2014 were reviewed. Subjects were included in the analysis if they were at least 20 years old, required HD and had blood cultures yielding MRSA isolates.

### Data collection and case definitions

We collected data on patient demographics, clinical characteristics, co-morbidities, the Charlson co-morbidity index, healthcare-associated (HA) risk factors in the 12 months preceding the MRSA-positive culture, disease severity, length of hospitalization, infection foci, empiric and definitive therapies, vancomycin trough level (if available), laboratory drug susceptibility results and outcomes.

The HA risk factors for MRSA infections are defined as follows: (1) prior hospitalization, (2) indwelling percutaneous devices or catheters at the present admission, (3) receiving surgery, (4) long-term care facility or nursing home residence, and (5) MRSA colonization or infection within one year before bacteremia onset [12]. The severity of bacteremia was graded as follows: no sepsis or simple sepsis, severe sepsis, and septic shock [13]. Thrombocytopenia was defined as a platelet count <100,000 platelets/mm^3^, as determined by a complete blood cell count. The severity of sepsis at initial presentation was assessed by the Pitt score. The empirical antibiotic was defined as the antibiotic administered within the first 48 h of index blood culture collection. Antibiotic therapies were considered effective if the isolate was susceptible to at least one of the antibiotics administered according to the 2010 CLSI guidelines [14]. The vancomycin trough level was measured after at least 2 doses of vancomycin [15], and inadequate vancomycin therapy was defined as a vancomycin trough level of <15 mg/L [16]. The concomitant use of β-lactam was defined as β-lactams administration for ≥24 h concurrent with intravenous vancomycin [17].

The primary outcome was treatment failure, which was defined in terms of the following events: (1) 30-day mortality; (2) persistent bacteremia, defined as a positive blood culture for MRSA obtained after >7 days of effective antibiotic therapy; or (3) recurrent MRSA bacteremia within 30 days of discontinuation of anti-MRSA therapy.

### Classification of MRSA bloodstream infections

Patients with at least one positive MRSA blood culture were included in the study. Data collection and genotyping were conducted only on the first blood isolate from the same patient. Cases were identified as HO by the CDC epidemiologic definitions if the culture-positive sample was obtained > 48 h after admission and as CO if the culture-positive sample was obtained ≤ 48 h after admission [3, 4]. Strains carrying SCC*mec* element type I, II or III are defined as HA genotypes, and those harboring SCC*mec* type IV or V are defined as CA genotypes [6, 7]. The MRSA isolates were classified into two subgroups according to vancomycin minimal inhibitory concentrations (MICs): low (<1.5 mg/L) or high (≥1.5 mg/L).

### Microbiological and molecular characteristics of MRSA isolates

MRSA isolates were collected from the microbiology laboratory of Kaohsiung Medical University Hospital at the time of blood culture identification and stored at -80°C. All *S*. *aureus* isolates were identified and tested for susceptibility to antimicrobial agents using the Vitek 2 system (bioMeriéux, Marcyl’Etoile, France). The interpretation of susceptibility followed Clinical and Laboratory Standards Institute (CLSI) guidelines [14]. Vancomycin MICs were determined by the Vancomycin E-test (AB Biodisk, Solna, Sweden) using a 0.5 McFarland inoculum streaked evenly with a swab onto Mueller-Hinton agar plates [18].

A multiplex polymerase chain reaction (PCR) method was used for SCC*mec* typing, and multilocus sequence typing (MLST) was performed as described in previous studies [19, 20]. MLST and SCC*mec* types were further inferred for all strains. The PCR method described by Gilot P. *et al*. was used to determine accessory gene regulator (*agr*) typing [21].

### Statistical analysis

Categorical variables were compared by a chi-square test or Fisher’s exact test. Continuous variables were compared by Student’s *t*-test or the Mann-Whitney *U*-test, as appropriate. The proportions of CO-MRSA and CA genotypes over 6 years were examined using the linear regression model for linear trends. Variables with a *p*-value less than 0.05 in the univariate analysis were incorporated into a multivariate analysis. Of the many parameters that represent the initial clinical severity of illness, only the Pitt score was included in the multivariable analysis to avoid multicolinearity. A multivariate backward logistic regression was performed to evaluate the independent effects on the primary outcome after adjustments for baseline discrepancies between groups. A *p*-value less than 0.05 was considered significant. The analyses were performed using SPSS v19 (SPSS Inc., Chicago, IL, USA).

## Results

### Clinical characteristics of the CO- and HO-MRSA groups

A total of 106 patients with MRSA bacteremia were identified during the study period. CO-MRSA infections occurred in 76 patients (71.7%), and 30 patients (28.3%) had HO- MRSA infections. The clinical characteristics of CO- and HO-MRSA infections are shown in Table 1. Age, weight, and the percentages of co-morbidities were similar in both groups. A significantly higher percentage of patients with HO-MRSA infections had received a shorter duration of HD at the time of bacteremia. Most of the underlying diseases among both groups were similar, but a significantly higher percentage of patients with CO-MRSA bacteremia had indwelling percutaneous devices or catheters at admission. Most catheters were non-cuffed tunneled catheters. The initial presentation in patients with HO-MRSA bacteremia was more severe (higher percentages of severe sepsis/shock, ICU admission, Pitt score) than in patients with CO-MRSA bacteremia. The majority of infection foci in both groups were catheter-related infections, arteriovenous fistula/graft infections and skin and soft tissue infections. No significant difference was observed with regard to antimicrobial treatment, including the percentage of effective empiric, definitive antibiotics and concomitant therapies, between the groups. Catheter removal was performed in 39 of 51 (76.5%) patients with catheter-related infection. Arteriovenous fistulas or grafts were removed in 16 of 23 (69.6%) patients with an arteriovenous fistula/graft infection. The rates of catheter and arteriovenous fistula or graft removal were similar in both groups (S1 Table). No significant difference was observed with regard to the rate of source removal and time to remove an infected device for most infection foci between CO- and HO-MRSA groups, except for skin and soft tissue infection (S1 Table). However, there was no significant difference in treatment failure rates between the two groups.

**Table 1:** Demographic data, clinical features, molecular characteristics and therapeutic characteristics between healthcare-associated community onset (CO)- and healthcare-associated hospital onset (HO)- methicillin-resistant *Staphylococcus aureus* (MRSA) infections in hemodialysis patients.

	CO-MRSA (n = 76)	HO-MRSA (n = 30)	*P* value
Male	36 (47.4)	15 (50.0)	0.807
Age, mean ± SD	67.93 ± 12.71	68.57 ± 13.27	0.820
Weight (kg)	55.30 ± 10.61	52.68 ± 10.98	0.265
**Duration of dialysis, year**			
Mean ± SD	3.22 ± 4.51	1.76 ± 4.14	0.127
<1 year	31 (40.8)	24 (80.0)	**0.000**
**Types of vascular access**^a^			
NCTC	28 (36.8)	17 (56.7)	0.063
AVG	16 (21.1)	2 (6.7)	0.090
AVF	15 (19.7)	8 (26.7)	0.436
CTC	17 (22.4)	2 (6.7)	0.089
**Medical history**			
Diabetes	47 (61.8)	22 (73.3)	0.264
Congestive heart failure	18 (23.7)	7 (23.3)	0.969
Hypertension	47 (61.8)	16 (53.3)	0.422
Cardiovascular disease	23 (30.3)	10 (33.3)	0.758
Cancer	10 (13.2)	7 (23.3)	0.198
**Charlson comorbidity score**	4.97±1.86	5.57±1.59	0.128
**HA risk factors**			
Hospitalization	65 (85.5)	27 (90.0)	0.753
Use of percutaneous devices or catheters	39 (51.3)	22 (73.3)	**0.039**
Surgery	40 (52.6)	11 (36.7)	0.138
Long-term-care facility residence	29 (38.2)	11 (36.7)	0.887
Previous MRSA colonization/infection	11 (14.5)	6 (20.0)	0.485
Median vancomycin MIC (mg/liter [IQR])	1.0(0.75–1.5)	1.5 (1.0–1.5)	0.055
≥1.5	30 (39.5)	17 (60.7)	0.054
SCC*mec* types^b^			**0.001**
I, II or III	26 (38.2)	19 (76.0)	
IV, V	42 (61.8)	6 (24.0)	
Major MLST types (%)	ST59 (17.1)	ST239 (33.3)	0.157
	ST239 (17.1)	ST900 (16.7)	
Major *agr* type (%)	Type 1 (75%)	Type 1 (91.3%)	0.202
**Initial presentation**			
Thrombocytopenia	17 (22.4)	12 (40.0)	0.067
Severe sepsis/shock	20 (26.3)	13 (43.3)	0.088
Pitt score, mean ± SD	2.25 ± 1.39	3.20 ± 1.79	**0.004**
ICU admission	19 (25.0)	20 (66.7)	**0.000**
**Infection foci**			
Skin and soft tissue	8 (10.5)	3 (10.0)	1.000
Catheter-related infection	29 (38.2)	22 (73.3)	**0.001**
Arteriovenous fistula/graft infection	21 (27.6)	2 (6.7)	**0.019**
Endocarditis	8 (10.5)	1(3.3)	0.440
Orthopedic infection	5 (6.6)	0 (0)	0.318
Other/unknown infection sites	5 (6.6)	2 (6.7)	1.000
Effective empiric antibiotics^d^	46 (60.5)	15 (50)	0.323
Definitive effective antibiotics	73 (96.1)	28 (93.3)	0.712
Vancomycin	65 (85.5)	26 (86.7)	
Other anti-MRSA agents	8 (10.5)	2 (6.7)	
Mean vancomycin trough^e^	16.37 ± 5.93	13.26 ± 4.83	0.067
Inadequate vancomycin therapy^f^	14 (33.3)	9 (56.3)	0.111
Concomitant use of ß-lactam antibiotics^g^	34 (44.7)	18 (60.0)	0.157
Time to remove infected source, days^h^	4.94 ± 6.57	4.76 ± 4.47	0.912
**Outcome**			
Treatment failure	32 (42.1)	17 (56.7)	0.176
Persistent bacteremia	13 (17.1)	5 (16.7)	1.000
30-days mortality	18 (23.7)	13 (43.3)	**0.045**
Recurrent bacteremia	19 (25.0)	6 (20.0)	0.585

AVF, arteriovenous fistula; AVG, arteriovenous graft; CTC, cuffed tunneled catheter; HA, healthcare-associated; MRSA, methicillin-resistant *staphylococcus aureus*; NCTC, non-cuffed tunneled catheter; SD, standard deviation; TC, tunneled catheter

^a^ one instance of missing data in the HO-MRSA group

^b^ data for 96 (90.6%) isolates were available for SCC*mec* typing, 3 isolates were untypable

^c^ data for 96 (90.6%) isolates were available for *agr* typing, 13 isolates were untypable.

^d^ defined as the intravenous administration of vancomycin, teicoplanin, daptomycin, or linezolid within 48 h of obtaining the index blood culture

^e^ vancomycin trough levels were tested in 58 patients, 42 in the CO-MRSA group and 16 in the HO-MRSA group.

^f^ defined as a vancomycin trough level of < 15 mg/L measured after the 3rd dialysis program.

^g^ ß -lactam antibiotics, including ampicillin/sulbactam, piperacillin/tazobactam, ceftriaxone, ceftazidime, cefepime, ertapenem, imipenem, and meropenem

^h^ first positive blood culture for MRSA as the index date

### Molecular characteristics of the CO- and HO-MRSA groups

SCC*mec* genotyping and MRSA MLST typing were available for 96 MRSA isolates (90.6% of cases). Among the 96 isolates, thirty-eight (39.6%) isolates were SCC*mec* type III, twenty-five (26.0%) were SCC*mec* type V, twenty-three (24.0%) were SCC*mec* type IV, six (6.3%) were SCC*mec* type II, one (1.0%) was SCC*mec* type I and three (3.1%) isolates were untypable. The molecular characteristics of CO- and HO-MRSA isolates are presented in Table 1. Significantly different proportions of SCC*mec* genotypes were observed between HO- and CO-MRSA isolates. Most HAHO-MRSA isolates were HA-genotypes (76.0%), and most HACO-MRSA isolates were CA-genotypes (61.8%).

**Fig 1:**
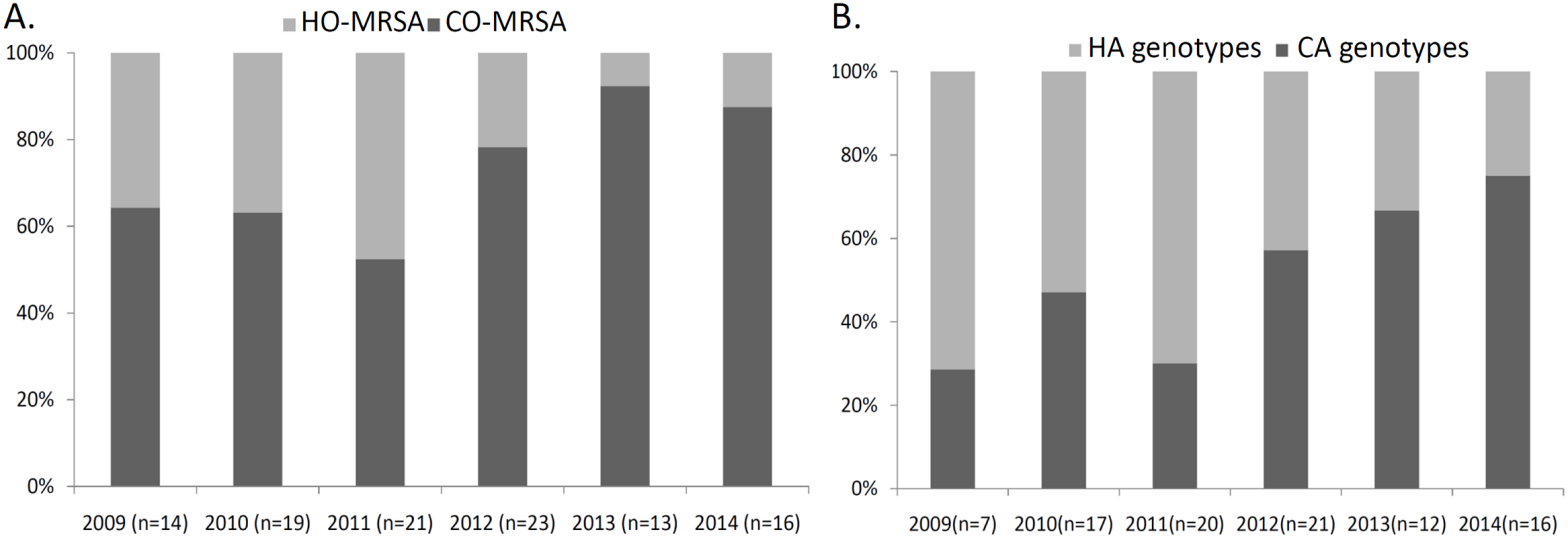
(A) The proportions of healthcare-associated community onset (CO)- and healthcare-associated hospital onset (HO)-MRSA bacteremia in hemodialysis cases in a Taiwanese medical center from 2009 to 2014. A linear trend toward an increasing proportion of CO-MRSA during 2009 to 2014 was observed (R^2^ = 0.662, *p* = 0.062). (B) The proportions of CA genotypes (SCC*mec* type IV, V) and HA genotypes (SCC*mec* type I, II or III strains) in hemodialysis cases in a Taiwanese medical center from 2009 to 2014. A linear trend toward an increasing proportion of CA genotypes during 2009 to 2014 was observed (R^2^ = 0.791, *p* = 0.018).

The proportions of HO- and CO-MRSA infections for each year of the study are presented in Fig 1A. The differences in SCC*mec* types by year are shown in Fig 1B. The proportion of CO-MRSA increased over time (*p* = 0.062). The proportion of CA genotypes significantly increased with time (*p* = 0.018).

ST239 was the most common MLST type. It accounted for 23 (23.9%) isolates. ST59 accounted for 17 (17.7%) isolates, and ST45 accounted for 13 (13.5%) isolates. The remaining isolates belonged to various other ST types. ST239 was identified in 33.3% of HO-MRSA cases and in 17.1% of CO-MRSA cases. ST45 and ST59 were identified in 20.0% of HO-MRSA cases and in 31.5% of CO-MRSA cases. The genotype analysis of the *agr* gene revealed that most isolates belonged to *agr* type 1 (Table 1).

### Outcome associations with the SCC*mec* genotypes

The main clinical characteristics of the MRSA bacteremia cases infected with HA and CA genotypes were not significantly different (S2 Table). The numbers of HA risk factors were not significantly different for cases of both groups (*p* = 0.243). Compared with cases with CA genotypes, cases with HA genotypes showed more severe manifestations relative to the proportions of severe sepsis/shock and higher Pitt scores. There was no significant difference in antimicrobial treatment administration for cases of both groups, including the percentage of effective empiric, definitive antibiotics and concomitant therapies. No significant difference was observed with regard to the rate of source removal and time to remove infected devices between CA and HA genotypes (S3 Table).

SCC*mec* type III MRSA isolates had the highest ratio of high vancomycin MIC (2 mg/L) (9/38, 23.7%), followed by SCC*mec* type II (1/6, 16.7%), SCC*mec* type V (1/25, 4%) and 0% in SCC*mec* type IV isolates. The main MLST type of MRSA with SCC*mec* type III isolates was ST239, the main MLST type with SCC*mec* type IV was ST59, and the main MLST type with SCC*mec* type V was ST45.

**Fig 2:**
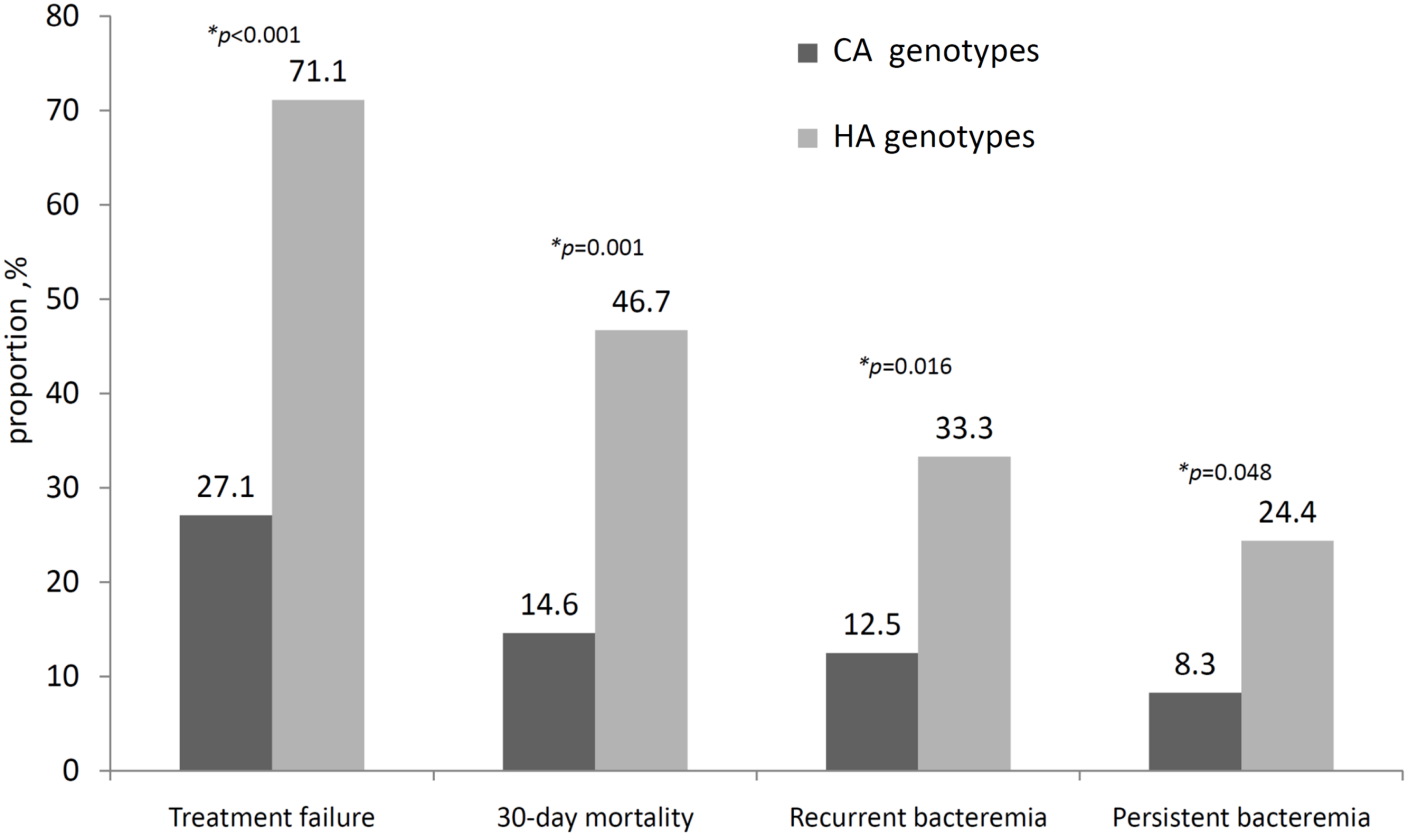
Treatment outcomes of 93 hemodialysis patients with community-associated, healthcare-associated methicillin-resistant *Staphylococcus aureus* bacteremia by genotypes.

Treatment failure was significantly lower in patients with CA genotypes of MRSA bacteremia in the univariate analysis (OR, 0.15; 95% CI, 0.06–0.37; *p* <0.001). This association was driven by differences between treatment groups with regard to 30-day mortality (OR, 0.20; 95% CI, 0.07–0.53; *p* = 0.001), persistent bacteremia (OR, 0.28; 95% CI, 0.08–0.96; *p* = 0.048) and recurrent bacteremia (OR, 0.29; 95% CI, 0.10–0.82; *p* = 0.016) (Fig 2).

### Predictors of treatment failure

In the study, 49 (46.2%) cases had treatment failure outcomes. There was no significant difference in the primary outcome between patients with HO-MRSA infections versus CO-MRSA infections (OR, 1.80; 95% CI, 0.77–4.22; *p* = 0.176). In the univariate analysis, cuffed tunneled catheter use, underlying cardiovascular diseases, a history of MRSA colonization or infection, initially severe clinical presentation, infective endocarditis, high vancomycin MICs and SCC*mec* type were associated with treatment failure (Table 2). The multivariate analysis revealed that a higher Pitt score was associated with treatment failure (OR:1.81; 95% CI, 1.23–2.67; *p* = 0.003) and CA genotypes (OR: 0.18; 95% CI, 0.07–0.49; *p* = 0.001) were associated with better primary outcomes (Table 3).

**Table 2:** Univariate analyses of the association between potential predictor variables and treatment failure in patients with methicillin-resistant *Staphylococcus aureus* (MRSA) bacteremia.

Variable	Odds Ratio (95% CI)	*p* value
Age, per year increase	1.03(0.99–1.06)	0.105
Weight, per kg increase	1.00(0.97–1.04)	0.935
Duration of dialysis, year	0.98(0.90–1.07)	0.696
Duration of dialysis, < 1 year	1.69(0.78–3.65)	0.183
NCTC	0.47(0.21–1.03)	0.060
AVG	1.57(0.57–4.36)	0.386
AVF	0.69(0.27–1.77)	0.442
CTC	3.07(1.07–8.84)	0.038
Diabetes	1.20(0.54–2.69)	0.652
Congestive heart failure	0.46(0.18–1.18)	0.107
Hypertension	0.61(0.28–1.34)	0.217
Cardiovascular disease	2.33(1.01–5.41)	0.048
Cancer	0.58(0.20–1.72)	0.327
Charlson comorbidity score	0.98(0.79–1.21)	0.834
Hospitalization	3.67(0.96–14.01)	0.057
Previous MRSA colonization/infection	3.37(1.10–10.39)	0.034
Surgery	1.97(0.91–4.28)	0.086
Use of percutaneous devices or catheters	0.83 (0.38–1.80)	0.637
Long-term-care facility residence	1.28(0.58–2.81)	0.544
HO-MRSA	1.80 (0.77–4.22)	0.178
Thrombocytopenia	1.99(0.84–4.74)	0.119
Severe sepsis/shock	6.38(2.51–16.24)	0.000
Pitt score, per increase	2.03(1.43–2.88)	0.000
ICU admission	4.60(1.97–10.77)	0.000
Skin and soft tissue infection	1.45(0.41–5.08)	0.560
Catheter-related infection	0.76(0.52–1.12)	0.165
Arteriovenous fistula/ graft infection	0.69(0.27–1.77)	0.442
Endocarditis	10.93(1.32–90.81)	0.027
Orthopedic infection	1.79(0.29–11.20)	0.532
Other/unknown infection sites	0.86(0.18–4.06)	0.853
Effective empiric antibiotics	0.97(0.45–2.10)	0.938
Definitive effective antibiotics	0.20(0.02–1.86)	0.158
Inadequate vancomycin therapy	0.68(0.23–1.98)	0.480
Concomitant use of ß-lactam antibiotics	1.83(0.85–3.97)	0.124
Time to remove infected source, days	1.05(0.97–1.15)	0.229
Vancomycin MIC≥1.5	3.53(1.57–7.94)	0.002
CA genotypes	0.15(0.06–0.37)	0.001

AVF, arteriovenous fistula; AVG, arteriovenous graft; CTC, cuffed tunneled catheter; HTN, hypertension; HO, hospital associated hospital onset; MIC, minimal inhibitory concentration; MRSA, methicillin-resistant *staphylococcus aureus*; NCTC, non-cuffed tunneled catheter; TC, tunneled catheter; 95% CI, 95% confidence interval

**Table 3:** Multivariate analyses of the association between potential predictor variables and treatment failure in patients with methicillin-resistant *Staphylococcus aureus* (MRSA) bacteremia.

	Univariate analysis		Multivariate analysis					
			All patients (n = 106)		vancomycin MICs<1.5 (n = 57)		vancomycin MICs ≥1.5 (n = 47)	
Variable	Odds Ratio (95% CI)	*p* value	Odds Ratio (95% CI)	*p* value	Odds Ratio (95% CI)	*p* value	Odds Ratio (95% CI)	*p* value
CTC	3.07(1.07–8.84)	0.038	…		8.09(1.32–49.73)	0.024	…	
Cardiovascular disease	2.33(1.01–5.41)	0.048	…		5.29(0.91–30.78)	0.064	…	
Previous MRSA colonization/infection	3.37(1.10–10.39)	0.034	…		…		…	
Pitt score, per increase	2.03(1.43–2.88)	0.000	1.81(1.23–2.67)	0.003	1.70(0.93–3.09)	0.084	1.76(1.02–3.05)	0.043
Endocarditis	10.93(1.32–90.81)	0.027	10.31(0.90–117.95)	0.061	…		…	
Vancomycin MIC ≥1.5^a^	3.53(1.57–7.94)	0.002	…		NE		NE	
CA genotypes	0.15(0.06–0.37)	0.000	0.18(0.07–0.49)	0.001	0.03(0.003–0.26)	0.001	…	

CTC, cuffed tunneled catheter; MIC, minimal inhibitory concentration; MRSA, methicillin-resistant *staphylococcus aureus*; NE, not entered into logistic regression model; 95% CI, 95% confidence interval

^a^ two instances of missing vancomycin MIC data

We further explored whether the vancomycin MIC level differences between different SCC*mec* type MRSA isolates accounted for the disparity in clinical impacts on MRSA bacteremia patients. A subgroup analysis stratified by vancomycin MIC was conducted to clarify this possible confounding effect. In the group with a vancomycin MIC <1.5 mg/L (n = 57), the multivariate analysis showed that cuffed tunneled catheter use was associated with treatment failure (OR: 8.09; 95% CI, 1.32–49.73; *p* = 0.024) and that CA genotypes (OR: 0.03; 95% CI, 0.003–0.26; *p* = 0.001) were associated with better primary outcomes. In the group with a vancomycin MIC ≥1.5 mg/L (n = 47), the only risk factor for treatment failure was Pitt score (OR: 1.76; 95% CI, 1.02–3.05; *p* = 0.043) (Table 3). The association between CA genotypes and treatment failure was consistently seen in subgroup analyses, except for patients with Pitt score ≥2 (OR: 0.20; 95% CI, 0.03–1.19; *p* = 0.076) (Table 4).

**Table 4:** Subgroup analysis of the association between potential predictor variables and treatment failure in patients with methicillin-resistant *Staphylococcus aureus* (MRSA) bacteremia.

	Multivariate analysis for subgroups							
	Pitt score ≤ 2 (n = 71)		Pitt score > 2 (n = 35)		CO-MRSA (n = 76)		HO-MRSA (n = 30)	
Variable	Odds Ratio (95% CI)	*p* value	Odds Ratio (95% CI)	*p* value	Odds Ratio (95% CI)	*p* value	Odds Ratio (95% CI)	*p* value
CTC	…		…		…		…	
Cardiovascular disease	3.25 (0.87–12.22)	0.081	…		13.63 (2.35–79.10)	0.004	0.06 (0.004–0.81)	0.035
Previous MRSA colonization/infection	…		…		…		…	
Pitt score, per increase	NE		NE		2.86 (1.39–5.90)	0.004	…	
Endocarditis	8.93 (0.73–109.10)	0.086	…		9.73 (0.65–145.51)	0.099	…	
Vancomycin MIC≥1.5	…		…		…		…	
CA genotypes	0.14 (0.04–0.50)	0.002	0.20 (0.03–1.19)	0.076	0.08 (0.02–0.40)	0.002	0.06 (0.004–0.81)	0.035

CTC, cuffed tunneled catheter; CO, hospital-associated community onset; HO, hospital-associated hospital onset; MIC, minimal inhibitory concentration; MRSA, methicillin-resistant *staphylococcus aureus*; NE, not entered into logistic regression model; 95% CI, 95% confidence interval

## Discussion

In this study, we identified an increasing trend of CO-MRSA in the HD population with MRSA bacteremia in a Taiwanese medical center during 2009–2014. Increasingly, MRSA bacteremia cases in the HD population in Taiwan are due to CA-genotypes, indicating the successful spread of CA genotypes in the HD population. This finding that CA- and HA-MRSA strains infected HD patients in healthcare facilities is similar to the situation of the co-existence of CA and HA genotypes in hospital settings [1, 22, 23]. Our results revealed that the CA genotype is a better predictor of clinical outcome than the epidemiologic classification of CO- or HO-infections. This strain-specific virulence factor effect was an independent risk factor for treatment outcomes in a subgroup analysis for patients with a vancomycin MIC < 1.5 mg/L, but this effect was attenuated with a vancomycin MIC ≥1.5 mg/L.

In Taiwan, ST59 is the major CA MLST type, and ST239 and ST45 are the most common MLST types for hospital-acquired infections and healthcare-associated infections, respectively [24, 25]. Our results revealed the existence of these three major MLST types in the HD population. We found increasing infections from CA genotypes in the HD population in Taiwan, a trend that matches the increase in CA genotypes observed in the HD population in USA [3].

It has been reported in the USA and in Taiwan that CA genotypes were independently associated with better treatment outcomes than HA genotypes in the general population [26, 27]. In the early 2000s, 80% of HD patients with MRSA bacteremia were infected with HA genotypes [2, 28, 29]. Only a few studies have evaluated the impact of CA or HA genotypes in the HD population. Earlier reports revealed that CA genotypes were not significantly associated with 30-day mortality [28, 29]. In this study, the characteristics of patients with HO-MRSA bacteremia included a shorter duration of HD, more severe clinical presentation, and more catheter-related/vascular access infections, but this epidemiological classification of MRSA infections (HO- or CO-MRSA) was not associated with treatment failure.

Strain differences were observed between HO-MRSA (76% HA genotypes and 24% CA genotypes) and CO-MRSA (38% HA genotypes and 62% CA genotypes). A study in the USA showed that patients who resided in long-term care facilities were less likely to harbor CA genotypes [30], but the current study did not demonstrate the importance of this factor in Taiwan. It has been reported that CA genotypes were more prevalent in patients with a lower number of HA risk factors [31]. However, the numbers of HA risk factors were not significantly different between groups with CA- or HA genotypes in the HD population in this study (*p* = 0.243). In Taiwan, CA genotypes have circulated in the healthcare setting; traditional distinctions between HA-MRSA and CA-MRSA strains based on clinical characteristics are becoming less useful [6, 7]. Our results support that the approach that differentiated CA- or HA-MRSA strains based on SCC*mec* type is of clinical significance.

We evaluated multiple factors, including patient comorbidities, clinical syndromes, clinical severity and therapeutic strategies, and demonstrated that CA genotypes were significantly associated with better clinical outcomes in the HD population (OR: 0.18; 95% CI, 0.07–0.49; *p* = 0.001). In this study, the vancomycin MICs were lower for CA-MRSA strains than for HA-MRSA strains. In the sub-analysis according to different vancomycin MICs, CA genotypes represented an independent risk factor for clinical outcomes among isolates with vancomycin MICs < 1.5 mg/L (OR: 0.03; 95% CI, 0.003–0.26; *p* = 0.001). The strain-specific differences in clinical outcomes have been suggested to be due to different intrinsic virulence factors [26, 27, 32]. Among isolates with vancomycin MICs ≥1.5 mg/L, the only significant factor for clinical outcome was clinical severity (OR: 1.76; 95% CI, 1.02–3.05; *p* = 0.043). The effect of CA genotypes on clinical outcomes was not observed with vancomycin MICs ≥1.5 mg/L.

Maintaining a vancomycin trough level within 15–20 mg/L to treat severe MRSA infections is recommended because a ratio of the vancomycin level area under the curve to the minimum inhibitory concentration of the *S*. *aureus* isolate (AUC/MIC) of > 400 can be targeted [16]. However, inadequate vancomycin therapy was not associated with treatment failure in this study. Because the value of the AUC may be significantly increased in the HD population, aggressively targeting a pre-HD level of 15–20 mg/L may not be needed to achieve an AUC/MIC ratio > 400 [33]. This may be the reason that 12% of the patients with low vancomycin trough levels in this study did not have poor clinical outcomes.

The limitations of this study include the following: first, an information bias may have been introduced due to the nature of the retrospective design. Second, this study was conducted in a single center in Taiwan, and the predominant CA genotypes in this study were ST59-SCC*mec* type IV and ST45-SCC*mec* type V. Whether our study findings can be generalized to different CA genotypes circulating in other HD populations in different geographic regions should be further evaluated.

In conclusion, these findings indicate that CA genotypes, but not the epidemiologic classification of CO-MRSA, impact the clinical outcome of MRSA bacteremia in the HD population. The difference in clinical impact between CA and HA genotypes was significant for isolates with vancomycin MICs < 1.5 mg/L.

## Supporting information

S1 Table Infected source removal rate in different infection foci between healthcare-associated community onset (CO)- and healthcare-associated hospital onset (HO)- methicillin-resistant *Staphylococcus aureus* (MRSA) infections in hemodialysis patients. (DOCX)

S2 Table Demographic data, clinical features, molecular characteristics and therapeutic characteristics between SCC*mec* type IV or V and SCC*mec* type I, II or III genotypes of methicillin-resistant *Staphylococcus aureus* (MRSA) infections in hemodialysis patients. (DOCX)

S3 Table Infected source removal rate in different infection foci between SCC*mec* type IV or V and SCC*mec* type I, II or III genotypes of methicillin-resistant *Staphylococcus aureus* (MRSA) infections in hemodialysis patients. (DOCX)
