# Supplementary material for: Association of bacterial genotypes and epidemiological features with treatment failure in hemodialysis patients with methicillin-resistant Staphylococcus aureus bacteremia
Source: PLoS One. 2018 Jun 4;13(6):e0198486. doi: 10.1371/journal.pone.0198486 (PMC5986133; doi:10.1371/journal.pone.0198486)
Supplement: S1 Table — (DOCX) [file pone.0198486.s001.docx]

| Infection foci | Removal source case numbers/ non-removal cases numbers | CO-MRSA (n=76) | HO-MRSA  (n=30) | *P* value |
| --- | --- | --- | --- | --- |
|  | Time to remove infected source, days ± standard deviation |  |  |  |
| Skin and soft tissue | | 5/3 | 2/1 | 1.000 |
|  |  | 1.0 ± 0.7 | 8.5 ± 6.4 | 0.044 |
| Catheter related infection | | 22/7 | 17/5 | 0.906 |
|  |  | 3.6 ± 5.1 | 4.1 ± 4.5 | 0.241 |
| Arteriovenous fistula/ graft infection | | 15/6 | 1/1 | 0.526 |
|  |  | 7.7 ± 9.0 | 7.0 | 0.826 |
| Endocarditis | | 3/5 | 1/0 | 0.444 |
|  |  | 7.7 ± 6.5 | 6.0 | 0.655 |
| Orthopedic infection | | 2/3 | 0/0 | N/A |
|  |  | 4.5 ± 5.0 |  | N/A |
| Other/unknown infection sites | | 1/4 | 0/2 | 1.000 |
|  |  | 5.0 |  | N/A |
| Total infection foci | | 47/29 | 21/9 | 0.430 |
|  |  | 4.94 ± 6.57 | 4.76 ± 4.47 | 0.912 |

**S1 Table.** Infected source removal rate in different infection foci between healthcare-associated community onset (CO)- and healthcare-associated hospital onset (HO)- methicillin-resistant *Staphylococcus aureus* (MRSA) infections in hemodialysis patients

N/A: not available

Categorical variables were compared by a chi-square test or Fisher’s exact test. Continuous variables were compared by Student’s *t*-test or the Mann-Whitney *U*-test as appropriate.
