# Supplementary material for: Association of bacterial genotypes and epidemiological features with treatment failure in hemodialysis patients with methicillin-resistant Staphylococcus aureus bacteremia
Source: PLoS One. 2018 Jun 4;13(6):e0198486. doi: 10.1371/journal.pone.0198486 (PMC5986133; doi:10.1371/journal.pone.0198486)
Supplement: S2 Table — (DOCX) [file pone.0198486.s002.docx]

**S2 Table.** Demographic data, clinical features, molecular characteristics and therapeutic characteristics between SCC*mec* type IV or V and SCC*mec* type I, II or III genotypes of methicillin-resistant *Staphylococcus aureus* (MRSA) infections in hemodialysis patients

|  | SCC*mec* IV or V (n=48) | SCC*mec* I, II or III (n=45) | *P* value |
| --- | --- | --- | --- |
| Male  Age, mean ± SD  Weight (kg)  **Duration of dialysis, year**  Mean ± SD  <1 year  **Types of vascular access**^a^  NCTC  AVG  AVF  CTC  **Medical history**  Diabetes  Congestive heart failure  Hypertension  Cardiovascular disease  Cancer  **Charlson comorbidity score**  **HA risk factors**  Hospitalization  Use of percutaneous devices or catheters  Surgery  Long-term-care facility residence  Previous MRSA colonization/infection  **Number of HA risk factors** | 19 (39.6)  68.21 ± 13.35  55.70 ± 10.14  3.66 ± 5.16  22 (45.8)  24 (50.0)  7 (14.6)  8(16.7)  9 (18.8)  29 (60.4)  11 (22.9)  26 (54.4)  16 (33.3)  9 (18.8)  5.00 ± 1.95  39 (81.3)  30 (62.5)  24 (50.0)  17 (35.4)  6 (12.5)  2.4 ± 1.0 | 22 (48.9)  69.58 ± 11.05  55.45 ± 10.80  2.47 ± 3.99  23(51.1)  13(28.9)  10 (22.2)  11 (24.4)  10 (22.2)  29 (64.4)  11 (24.4)  29 (64.4)  13 (28.9)  3 (6.7)  4.91 ± 1.56  41 (91.1)  25 (55.6)  25 (55.6)  20 (44.4)  10 (22.2)  2.7 ± 1.2 | 0.366  0.593  0.302  0.219  0.611  **0.040**  0.344  0.355  0.678  0.689  0.862  0.314  0.644  0.122  0.809  0.235  0.496  0.592  0.374  0.214  0.243 |
| Median vancomycin MIC (mg/liter [IQR])  ≥1.5  MLST of major genotype (%)  Major *agr* type (%) | 1.0(0.75-1.0)  6 (12.5)  ST59 (33.3)  ST45 (27.1)  type 1 (71.4%) | 1.5 (1.0-1.5)  32 (71.1)  ST239 (51.1)  ST900 (24.4)  type 1 (84.4%) | 0**.000**  0**.000**  0.158 |
| **Initial presentation**  Thrombocytopenia  Severe sepsis/shock  Pitt score, mean ± SD  ICU admission  **Infection foci**  Skin and soft tissue  Catheter related infection  Arteriovenous fistula/ graft infection  Endocarditis  Orthopedic infection  Other/unknown infection sites  Effective empiric antibiotics^b^  Definitive effective antibiotics  Vancomycin  Other anti-MRSA agents  Mean vancomycin trough^c^  Inadequate vancomycin therapy^d^  Concomitant use of ß-lactam antibiotics^e^  Time to remove infected source, days^f^  **Outcome**  Treatment failure  Persistent bacteremia  30-days mortality  Recurrent bacteremia | 13(27.1)  11 (22.9)  2.08 ± 1.24  10 (20.8)  2 (4.2)  25 (52.1)  14 (29.2)  3 (6.3)  1 (2.1)  3 (6.3)  32 (66.7)  47(97.9)  42 (87.5)  5 (10.4)  17.76 ± 6.64  8 (33.3)  20 (41.7)  4.39 ± 5.04  13 (27.1)  4 (8.3)  7 (14.6)  6 (12.5) | 14 (31.1)  20 (44.4)  3.11 ± 1.82  23 (51.1)  8 (17.8)  20 (44.4)  8 (17.8)  3(6.7)  4(8.9)  2 (4.4)  24 (53.3)  41(91.1)  36 (80.0)  5 (11.1)  15.21 ± 4.50  7 (30.4)  27 (60.0)  5.21 ± 6.94  32(71.1)  11 (24.4)  21 (46.7)  15 (33.3) | 0.669  **0.028**  **0.002**  **0.002**  **0.046**  0.461  0.197  1.000  0.194  1.000  0.189  0.194  0.133  0.831  0.077  0.596  **0.000**  0.048  0.001  0.016 |

AVF, arteriovenous fistula; AVG, arteriovenous graft; CTC, cuffed tunneled catheter; HA, healthcare associated; MRSA, methicillin-resistant *staphylococcus aureus*; NCTC, non-cuffed tunneled catheter; OR, Odds ratio; SD, standard deviation; TC, tunneled catheter; 95% CI, confidence interval.

^a^ one missing data in the SCC*mec* type I, II or III group

^b^ defined as the intravenous administration of vancomycin, teicoplanin, daptomycin, or linezolid within 48 h of obtaining the index blood culture

^c^ vancomycin trough levels data were available for 47 patients, including 23 in the group of SCC*mec* type I, II or III and 24 in the group of SCC*mec* type IV or V.

^d^ defined as a vancomycin trough level of < 10 ug/ml measured after the 3rd dialysis program.

^e^ ß -lactam antibiotics, including ampicillin/sulbactam, piperacillin/tazobactam, ceftriaxone, ceftazidime, cefepime, ertapenem, imipenem, and meropenem

^f^ First positive blood culture for MRSA as the index date
