# Supplementary material for: Association of bacterial genotypes and epidemiological features with treatment failure in hemodialysis patients with methicillin-resistant Staphylococcus aureus bacteremia
Source: PLoS One. 2018 Jun 4;13(6):e0198486. doi: 10.1371/journal.pone.0198486 (PMC5986133; doi:10.1371/journal.pone.0198486)
Supplement: S3 Table — (DOCX) [file pone.0198486.s003.docx]

**S3 Table.** Infected source removal rate in different infection foci between SCC*mec* type IV or V and SCC*mec* type I, II or III genotypes of methicillin-resistant *Staphylococcus aureus* (MRSA) infections in hemodialysis patients

| Infection foci | Removal source case numbers/ non-removal cases numbers | SCC*mec* IV or V (n=48) | SCC*mec* I, II or III (n=45) | *P* value |
| --- | --- | --- | --- | --- |
|  | Time to remove infected source, days ± standard deviation |  |  |  |
| Skin and soft tissue | | 1/1 | 5/3 | 1.000 |
|  |  | 13.0 | 1.8 ± 1.3 | 0.120 |
| Catheter related infection | | 20/5 | 13/7 | 0.258 |
|  |  | 3.2 ± 5.0 | 4.2 ± 3.7 | 0.131 |
| Arteriovenous fistula/ graft infection | | 10/4 | 6/2 | 1.000 |
|  |  | 5.9 ± 4.5 | 10.5 ± 13.1 | 0.546 |
| Endocarditis | | 1/2 | 2/1 | 1.000 |
|  |  | 8.0 | 3.5 ± 3.5 | 0.221 |
| Orthopedic infection | | 1/0 | 1/3 | 0.400 |
|  |  | 1.0 | 8.0 | 0.317 |
| Other/unknown infection sites | | 0/3 | 1/1 | 0.400 |
|  |  |  | 5.0 | N/A |
| Total infection foci | | 15/33 | 17/28 | 0.508 |
|  |  | 4.39 ± 5.04 | 5.21 ± 6.94 | 0.956 |

N/A: not available

Categorical variables were compared by a chi-square test or Fisher’s exact test. Continuous variables were compared by Student’s *t*-test or the Mann-Whitney *U*-test as appropriate.
